# Exploring single-head and multi-head CNN and LSTM-based models for road surface classification using on-board vehicle multi-IMU data

**DOI:** 10.1038/s41598-025-10573-2

**Published:** 2025-07-09

**Authors:** Luis A. Arce-Saenz, Javier Izquierdo-Reyes, Rogelio Bustamante-Bello

**Affiliations:** School of Engineering and Science, Tecnologico de Monterrey, Mexico City, 14380 Mexico

**Keywords:** Road Surface Condition Classification, Inertial Measurements, On-Board Sensing, Convolutional Neural Networks, Recurrent Neural Networks, Long Short-Term Memory, Civil engineering, Computer science

## Abstract

Accurate road surface monitoring is essential for ensuring vehicle and pedestrian safety, and it relies on robust data acquisition and analysis methods. This study examines the classification of road surface conditions using single- and multi-head deep learning architectures, specifically Convolutional Neural Networks (CNNs) and CNNs combined with Long Short-Term Memory (LSTM) layers, applied to data from Inertial Measurement Units (IMUs) mounted on vehicle’s sprung and unsprung masses. Various model architectures were tested, incorporating IMU data from different positions and utilizing both acceleration and angular velocity features. A grid search was conducted to fine-tune the architectures’ hyperparameters, including the number of filters, kernel sizes, and LSTM units. Results show that CNN+LSTM models generally outperformed CNN-only models. The highest-performing model, which used data from three IMUs in a single-head architecture, achieved a macro F1-score of 0.9338. The study highlights the effectiveness of combining IMU data in a single-head architecture and suggests that further improvements in classification accuracy can be achieved by refining the architectures and enhancing the dataset, particularly for more challenging road surface classes.

## Introduction

Road surface assessment is crucial for ensuring the safety of vehicles and pedestrians in urban areas. While traditional road assessment techniques are effective, advancements in technology have significantly enhanced the efficiency and accuracy of these tasks, allowing for faster and more reliable results. Additionally, the continuous development of artificial intelligence (AI) tools has led to the creation and implementation of even more insightful, rapid, and accurate road assessment techniques. Intelligent transportation systems (ITS) and smart cities increasingly rely on these advanced solutions to enable faster decision-making processes, which are essential for the success of modern ITS infrastructures and societies.

Non-traditional road assessment techniques primarily involve the ingestion and processing of data to extract meaningful insights, which are then translated into road condition information. Among the various data sources available, three stand out^1,2^: computer vision, laser scanning, and inertial measurements. These sources utilize vehicles to collect data as they travel over the roads. Each source offers unique advantages and disadvantages, as well as specific scenarios where they are most effective.

Computer vision techniques rely on cameras mounted on vehicles to capture images of the road surface. This approach allows for a comprehensive analysis of the road at a relatively low cost. However, it can be sensitive to environmental factors such as lighting conditions and the visibility of road markings, which may degrade image quality and affect road condition prediction accuracy^3–5^. Nevertheless, recent deep learning-based approaches, such as image-to-image translation, have shown potential in mitigating these limitations by converting poorly lit or nighttime images into daylight-like representations, thus improving robustness under varying illumination conditions^6^. Laser scanning, on the other hand, offers high precision and can capture detailed conditions of the road surface^7^, but it tends to be more expensive and requires highly specialized equipment to be deployed. Inertial measurement techniques are not dependent on weather or lighting conditions and can be easily installed on almost any vehicle. The data collection process is computationally lightweight, and the analysis is relatively simple, although typically less accurate and less detailed than the other two techniques^5^. A key limitation is that the vehicle must physically interact with road anomalies to detect them, and the output is usually limited to classification rather than fine-grained tasks such as semantic segmentation. Nevertheless, this limitation could be addressed by fusing inertial data with vision-based inputs to enable more detailed recognition and localization of defects.

This study focuses on optimizing the analysis of inertial measurement data to address the challenges of dense urban environments. In cities with extensive road networks and heavy traffic, there is a critical need for a road assessment solution that is not only cost-effective but also adaptable and precise, capable of handling the varying conditions of such complex settings. The foundation of this approach lies in analyzing time-series data from IMUs to detect and classify road conditions based on vehicle vibrations. This study aims to develop, implement, and test a deep learning (DL) framework that utilizes CNNs to classify time-series data from IMUs installed on both the sprung and unsprung masses of vehicles. The scope of the study encompasses the exploration and comparison of different CNN architectures, the integration of Recurrent Neural Network (RNN) layers, and the evaluation of various data input configurations to enhance the classification performance of the models.

The contributions of this study are as follows: (a) Development of deep learning models for road surface classification, leveraging convolutional and recurrent architectures (CNN-LSTM) to classify five distinct road conditions: even road, uneven road, manhole-pothole, speed bump, and pavement patch. (b) Analysis of sensor data configurations, comparing acceleration and angular velocity from both the sprung and unsprung masses to determine the optimal data fusion strategy for improved classification performance. (c) Performance evaluation and benchmarking, demonstrating the advantages of integrating LSTM layers for time-series classification and assessing the proposed models against existing approaches.

The remainder of this paper is organized as follows: The “Related Work” section provides an overview of existing research in road surface assessment techniques, focusing on on-board vehicle sensor-based methods. The “Methodology” section details the approach taken in this study, covering data collection, preprocessing, and the AI techniques employed for data analysis. The “Experiment setup” section describes the development of the proposed methodology. The “Results and analysis” section presents the metrics used to evaluate the classification performance of the AI models, and the corresponding analysis, discussing both the strengths and weaknesses of the trained models, as well as the experimental findings. Finally, the “Conclusion” section summarizes the study’s key outcomes and outlines potential directions for future research and improvements.

## Related work

Road surface monitoring (RSM) using non-traditional techniques has emerged from the need to accelerate road assessment processes while maintaining or exceeding the accuracy of traditional methods, such as the usage of profilometers or the development of condition indexes^8–10^ like the International Roughness Index (IRI). Traditional techniques, although precise, are often time-consuming and resource-intensive. Over the years, various studies have explored sampling techniques for assessing road surfaces, including computer vision and laser scanning, which offer millimeter-level precision in identifying small and large road distresses. Despite their proven accuracy and reliability in classifying and detecting road anomalies^3,5,7,11–13^, these methods are often costly and computationally demanding, limiting their scalability for widespread use or integration in multiple vehicles for mass data collection or crowd-sourced RSM tasks. Consequently, more affordable solutions, such as inertial measurement techniques, have gained recognition.

The inertial measurement approach captures acceleration and angular velocity data from vehicles as they travel across road surfaces. As vehicles pass over different road sections, the time-series data reflects peaks and irregularities corresponding to the road profile. It is important to note that sensor placement significantly influences both the quality and the characteristics of the data, as the response differs depending on whether the sensors are mounted on the sprung (damped) or unsprung (undamped) mass of the vehicle. This distinction affects the type of analysis that can be performed, as the nature of the data varies based on its source.

The RSM approach using inertial measurements has gained attention due to its simplicity, low cost, and minimal computational demands. Data can be collected via smartphones^14–21^ or IMUs^22–26^, strategically placed on vehicles. After collection, the data is preprocessed to enhance its quality, remove noise, and potentially enrich the dataset for more effective analysis. A variety of data analysis techniques have been applied to interpret the inertial data and identify corresponding road distress. Initial research focused on straightforward methods like threshold analysis^16,22,23^ or using Dynamic Time Warping techniques^17^ to detect road anomalies. Other studies aimed to calculate approximate road condition indexes, such as the IRI^14,24,26^, which provides a quantitative assessment of the overall condition of road segments. With the advancement of computational capabilities and the development of more sophisticated software tools, researchers began to apply machine learning (ML) techniques to classify and detect road distress in time-series data more effectively^15,18–20,27–29^. These ML approaches initially relied on traditional algorithms but have demonstrated notable improvements in classification accuracy and the range of road conditions that can be identified.

In recent years, there has been a shift toward DL techniques, particularly using CNNs^30,31^, which enable automatic feature extraction from raw sensor signals without the need for complex preprocessing. This approach has significantly improved classification performance by leveraging the inherent structure of time-series data. Additionally, some studies have incorporated RNNs into deep learning architectures^31,32^, allowing models to capture temporal dependencies within the data. These hybrid models have outperformed previous methodologies, both in terms of classification performance and the reduction of complex manual feature engineering efforts.

In summary, the field of RSM has evolved significantly from traditional methods to more advanced sensor-based techniques, leveraging both classical ML and DL approaches. While early methods focused on simpler analysis, the conception of CNNs and RNNs has brought about significant improvements in the accuracy and efficiency of these systems. Despite these advances, gaps remain in optimizing the classification performance and reducing computational costs for near real-time applications. This study builds on the foundation of previous work by further exploring DL architectures, specifically CNNs, and RNNs, to enhance the accuracy of road distress classification while also investigating the impact of different sensor configurations.

## Methodology

The proposed methodology consists of three main steps. First, data collection involves collecting inertial measurements from vehicles, with sampling conducted on both the sprung and unsprung masses. This dual sampling captures the vehicle’s response to the road profile, enriching the dataset with more comprehensive information on road conditions. Next, data preprocessing is performed to clean and prepare the raw data for analysis. This process includes applying various transformations and data curation techniques to create the final datasets for training the proposed DL models. Finally, the data processing phase details the design of the DL models for classifying the time-series data. This section describes the exploration of different model architectures and input configurations to achieve the best possible classification performance.

### Data collection

The data collection system was designed to capture inertial measurements from a vehicle’s sprung and unsprung masses, enabling AI models to extract a broader range of features related to road surface characteristics. This dual-source approach improves classification accuracy by leveraging distinct vehicle dynamic responses.

To build a robust dataset covering various road elements-including even and uneven surfaces, potholes, manholes, patches, and speed bumps-a custom data collection system^33^ was developed and deployed. Data was collected in Mexico City, where such road conditions are prevalent, using two test vehicles: a Chrysler Voyager and a Suzuki Vitara.

**Fig. 1:**
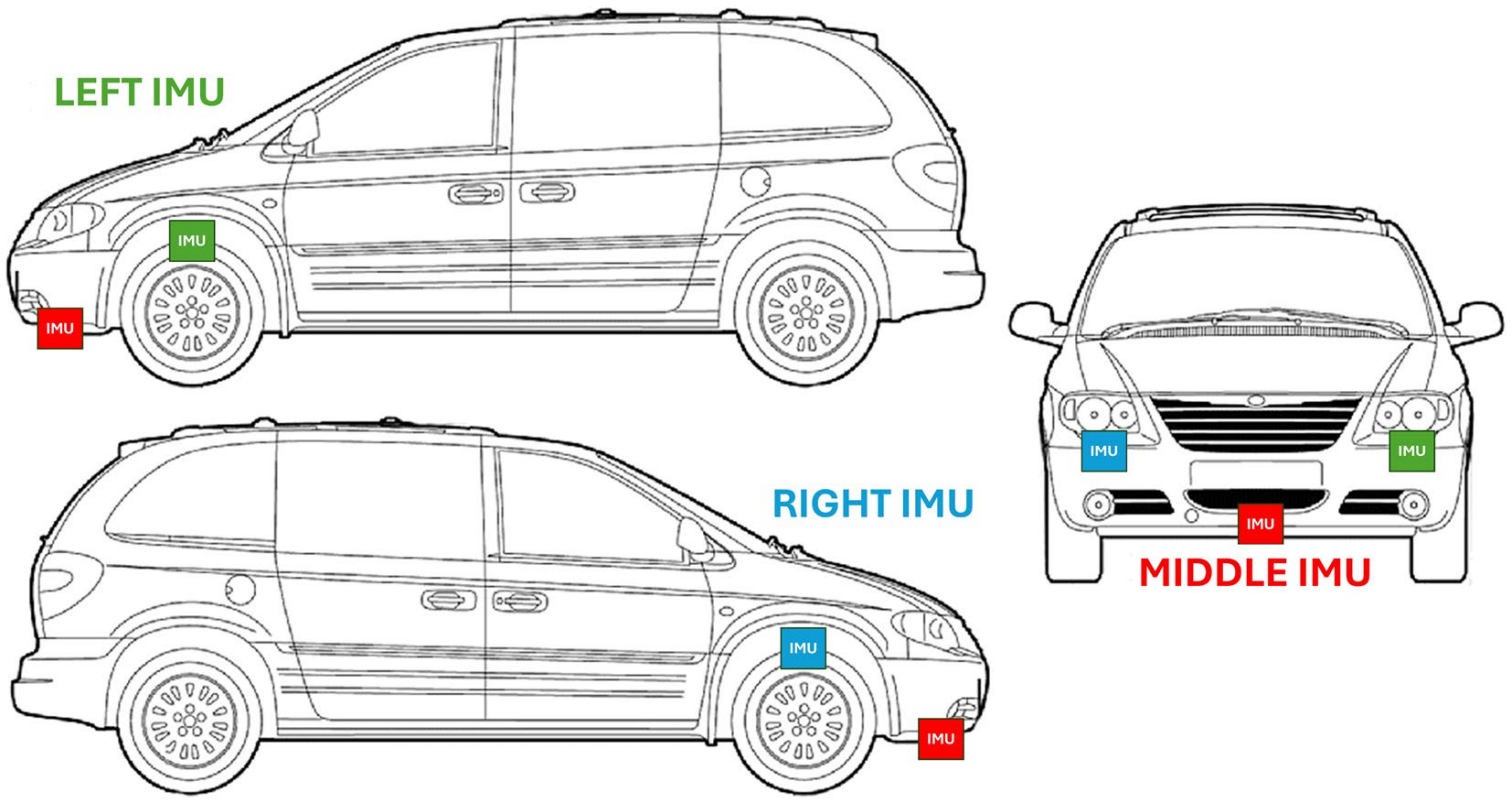
Arrangement of IMUs in the test vehicle, proposed in^33,34^. The Left and Right IMUs were attached to the joints of the shock absorbers, while the Middle IMU was installed at the front of the vehicle under the chassis.

Each vehicle was equipped with MPU-6050 IMUs connected to a Raspberry Pi 3 Model B+, logging acceleration and angular velocity data at 86 Hz. The IMUs were arranged as shown in Fig. 1. Road anomalies were manually annotated with timestamps, and a GPS antenna recorded their locations. The system continuously logged vibration data, geolocation, and labeled road conditions throughout each test run.

The inertial measurements were recorded as time-series data consisting of six features: acceleration and angular velocity along each of the three axes. The vehicle’s response varied depending on the type of road element encountered, reflecting the physical characteristics of the road surface.

**Fig. 2:**
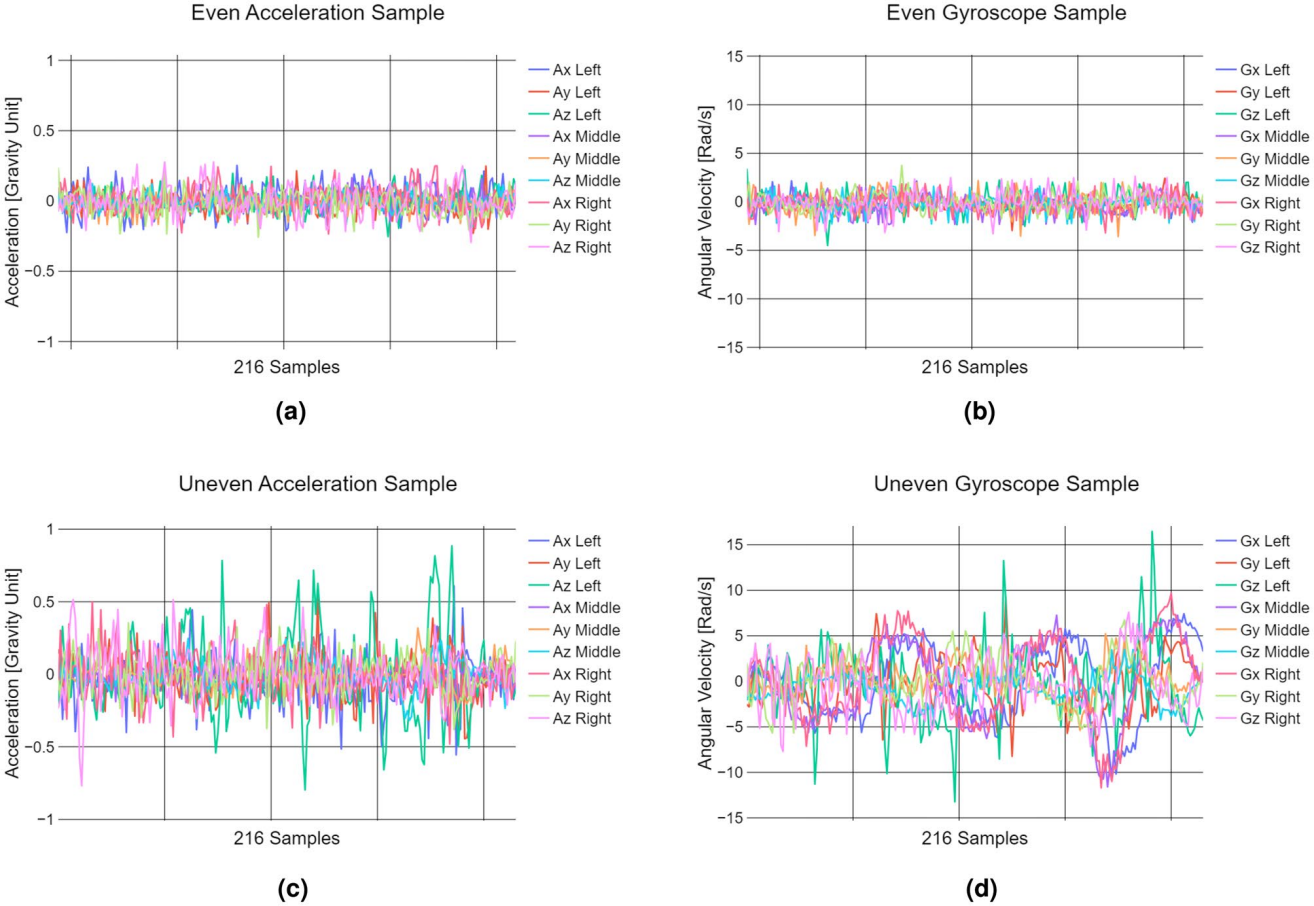
Data representation of the vehicle’s response over Even and Uneven roads. Even road **(a)** acceleration and **(b)** angular velocity. Uneven road **(c)** acceleration and **(d)** angular velocity.

Even road segments, defined as newly paved surfaces with no visible distress, exhibited minimal variations in sensor data. In contrast, uneven segments-including minor to major cracks, bumps from pavement patches, and small potholes-produced more pronounced acceleration and angular velocity responses. As shown in Fig. 2c, these surface irregularities led to noticeable excitations in acceleration, while subtle shifts in vehicle orientation were captured in angular velocity data (Fig. 2d). Figures 2a and 2b illustrate the contrasting stability of even road responses.

**Fig. 3:**
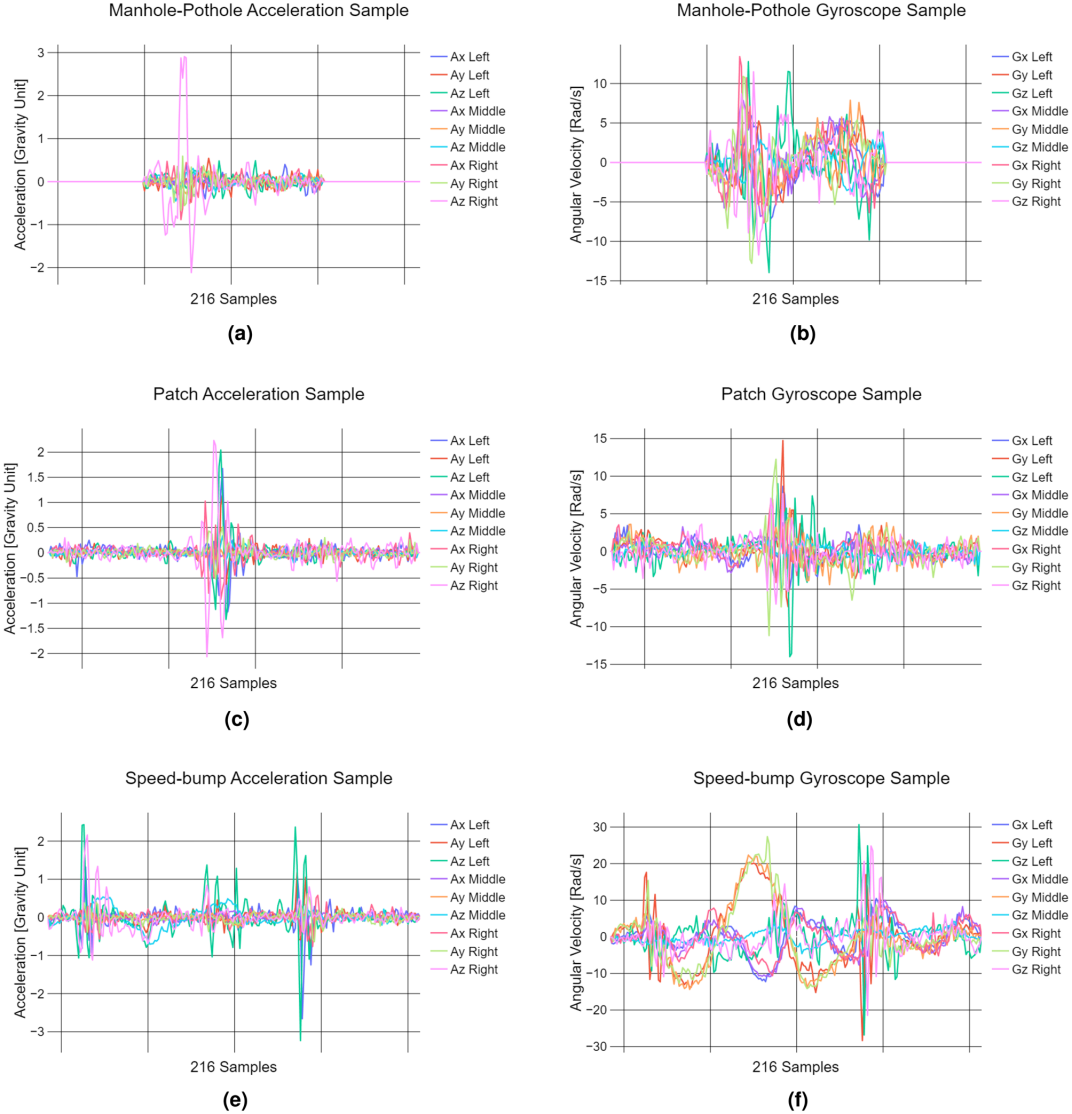
Data representation of road distresses. Manhole-Pothole **(a)** acceleration and **(b)** angular velocity. Patch **(c)** acceleration and **(d)** angular velocity. Speed bump **(e)** acceleration and **(f)** angular velocity.

Figure 3 illustrates the additional road surface classes analyzed in this study, each exhibiting distinct IMU response patterns. Manholes and potholes were grouped into a single class due to their similar geometry and vehicle interaction. As defined by ASTM International^10^, potholes are bowl-shaped depressions with sharp edges and near-vertical sides, resembling the structure of manhole covers commonly found in Mexico City. Figures 3a and 3b show the IMU response when the right tire encounters such a depression, resulting in a sudden acceleration spike followed by a distinct angular velocity change as the vehicle adjusts to the abrupt drop. Once past the depression, the acceleration stabilizes.

A similar acceleration pattern is observed for pavement patches, where both the wheels and chassis experience a sharp increase as the front tires make contact. Figures 3c and 3d illustrate this response, with wider patches affecting both tires simultaneously, while smaller patches may impact only one. Unlike manholes and potholes, the angular velocity stabilizes faster in this case.

The speed bump class exhibits a distinct response due to its raised structure spanning the entire road width. As the front tires make initial contact, acceleration spikes at both the wheels and chassis (Fig. 3e). Additional peaks occur along the Z-axis as the rear tires pass over. The corresponding angular velocity (Fig. 3f) reflects significant pitch changes, capturing the vehicle’s upward and downward motion.

The final dataset comprises 13,285 labeled instances across five road surface classes (Table 1), collected from both test vehicles. After acquisition, labels were refined through manual review of video recordings, and data was segmented into windows to ensure each event was fully captured.

**Table 1:** Count of labels identified during the data acquisition process.

**Labels**	**Count**
Even Road	9301
Uneven Road	2767
Manhole-Pothole	691
Speed bump	418
Patch	108
**Total**	**13285**

### Data preprocessing

Preprocessing was required to prepare the dataset for training. As shown in Table 1, the dataset was highly imbalanced, with two over-represented classes. To mitigate this, data augmentation was applied to generate additional samples and improve model performance.

**Fig. 4:**
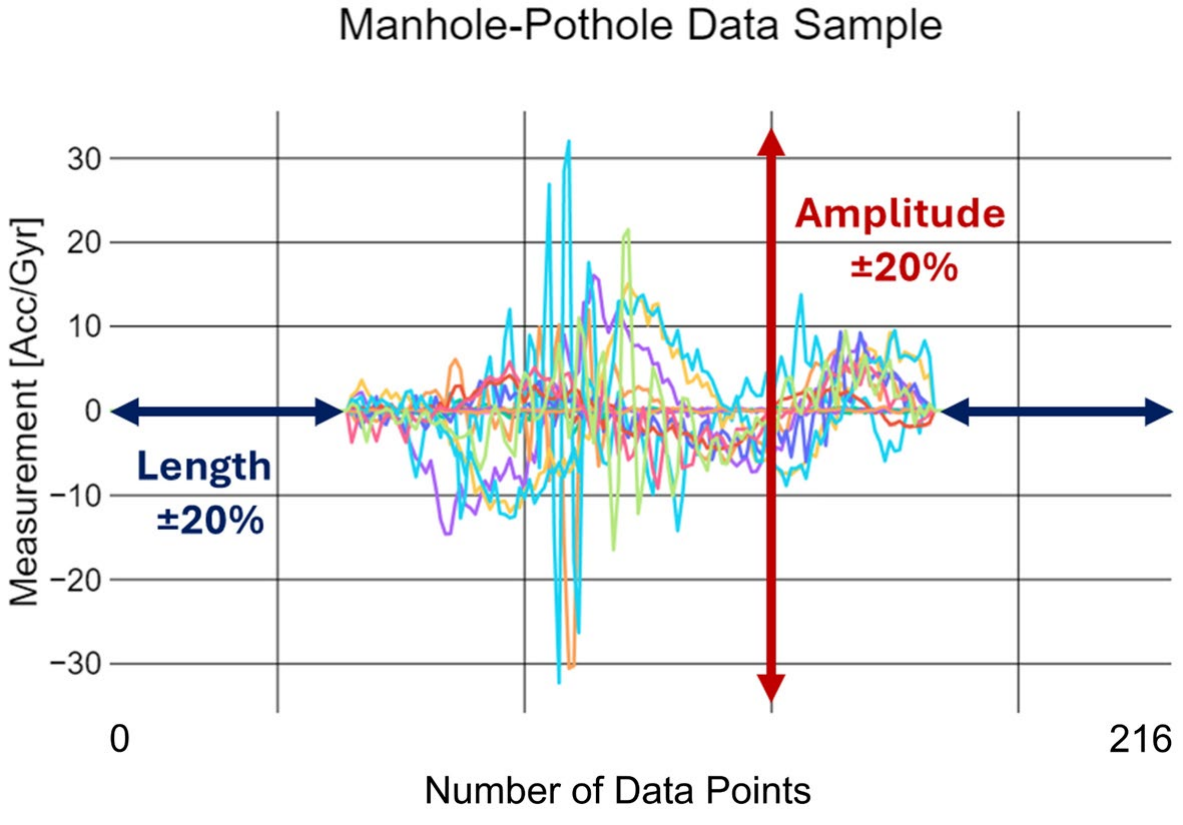
Example of a data window corresponding to the Manhole-Pothole label. This particular window contains significantly fewer data points than the median window length of 216 timestamps, illustrating the variability in event durations.

The data augmentation process, inspired by the methods described by Um et al.^35^, involved applying time-series transformations to generate synthetic samples. A random sample was selected and modified in amplitude and duration. As shown in Fig. 4, the amplitude was adjusted by ±20% to simulate variations in event intensity, while time warping altered the signal length by ±20% to account for differences in event duration.

While this method was effective and computationally efficient for the current study, we acknowledge the potential of using more advanced techniques, such as generative models. Approaches based on Generative Adversarial Networks (GANs) have shown promise in augmenting small datasets by generating synthetic samples that resemble real data, particularly in image-based tasks^36^. Moreover, recent studies have explored their application to time-series data augmentation as well^37^, providing new possibilities for enhancing vibration-based classification systems. However, such models often require larger datasets, architecture optimization, and significant computational resources. As such, their integration was considered beyond the scope of this work, but we propose it for future research.

To avoid overfitting and bias from excessive synthetic data, augmentation was limited to 1,000 samples per class for the “Patch,” “Speed bump,” and “Manhole-Pothole” labels. This cap helped maintain dataset diversity while ensuring underrepresented classes had sufficient examples for model training. The “Even” and “Uneven” labels, which already had adequate representation, were left unchanged. After augmentation, each class had 1,000 samples ensuring fair representation for training the models.

After balancing the dataset, further preparation was necessary to fit the fixed-length input requirements of the DL models. Given that the median window length before augmentation was 216 data points, samples with fewer points were zero-padded, with the original data centered in the padded sequence (see Fig. 4). After padding, the dataset was standardized to a mean of 0 and a standard deviation of 1. The final dataset, with 1,000 samples per class, was split into training, validation, and testing sets at a 70-20-10 ratio and saved as Numpy^38^ arrays for further processing.

### Data processing

**Fig. 5:**
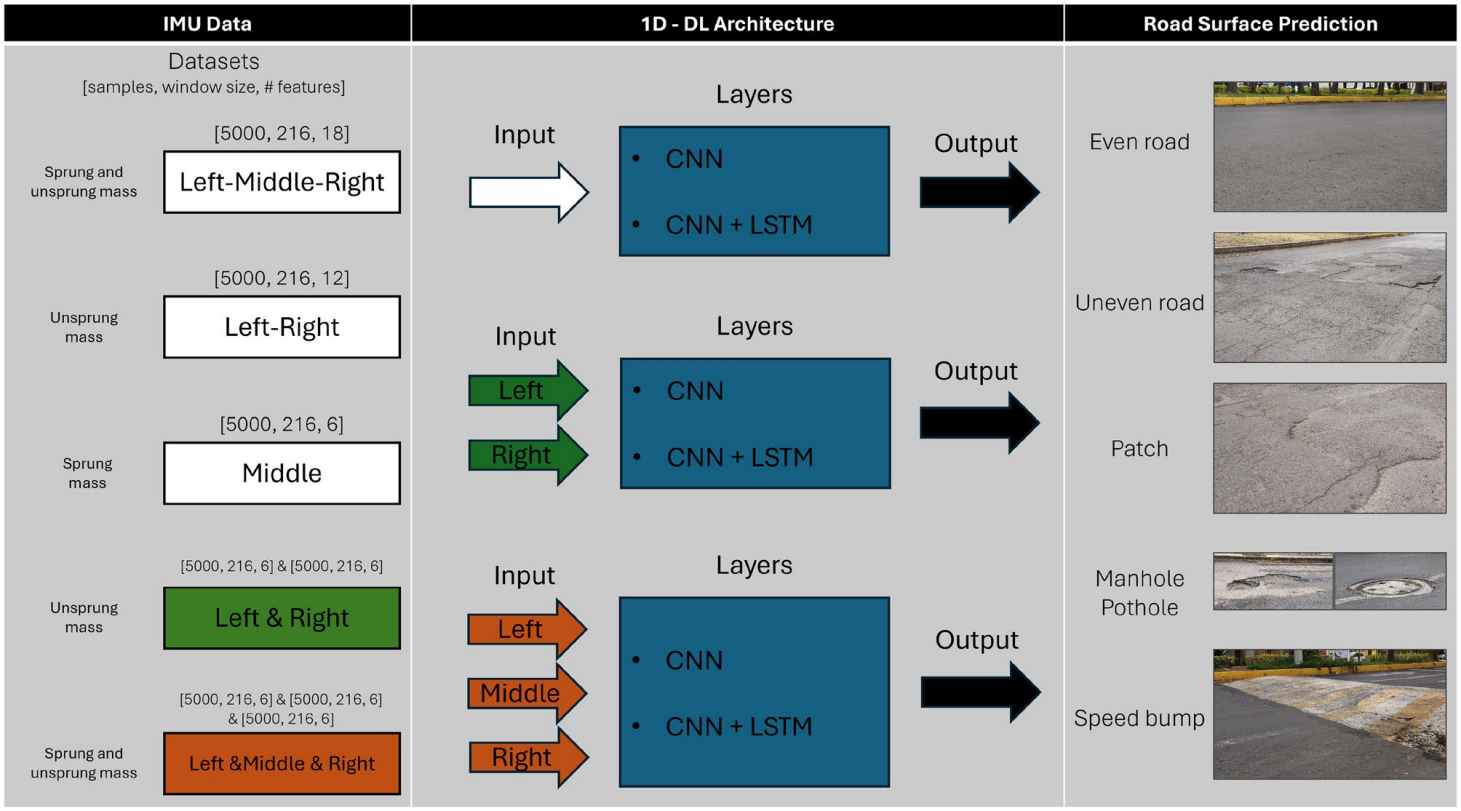
The figure shows the five datasets, their corresponding deep learning architectures, and the final output layer for classification. Each architecture has two variants: one using a pure CNN and the other incorporating LSTM layers.

The proposed approach divides the dataset into subsets to determine the most relevant data sources for classifying road surface conditions. As illustrated in Fig. 5 under the “IMU Data” section, five distinct datasets are introduced, categorized into three groups. The first group considers the combined IMU data as a single input for training the deep learning models. The second group evaluates the Left and Right IMUs separately, while the third treats all three IMUs-Left, Right, and Middle-as independent datasets.

Each IMU provides six features: acceleration and angular velocity along the X, Y, and Z axes. The models are trained and tested using both the full feature set and specific subsets, evaluating performance when using acceleration and angular velocity data separately. This results in three variations per dataset and architecture, allowing a more detailed analysis of each data component’s contribution to classification.

Each dataset is evaluated using two architectural approaches. The first employs a CNN-based architecture to assess whether convolutional layers alone can extract meaningful features from the sensor data for reliable classification. The second combines convolutional layers with RNN layers, specifically LSTM units, to capture both spatial and temporal patterns, potentially improving accuracy.

In total, thirty models were proposed based on all possible architecture and dataset combinations. The following subsections provide detailed descriptions of the proposed architectures.

#### Multi-head CNN

In recent years, DL solutions have emerged as state-of-the-art approaches for various data science challenges, outperforming traditional machine learning algorithms in many domains. CNNs, in particular, have shown strong performance in tasks involving structured data, including not only images and 3D point clouds but also 1D time-series signals.

CNNs use convolutional layers to apply filters that extract relevant patterns from the input data. While 2D convolutions are typically associated with image data, 1D convolutions are highly effective for analyzing time-series signals by scanning patterns along the temporal axis. In this study, we adopt 1D-CNNs to process raw vibration signals collected from the IMUs mounted on the vehicle. This approach eliminates the need for explicit feature engineering or complex processing techniques such as time-frequency transformations.

Although it is common to convert 1D time-series data into time-frequency representations (e.g., via Fourier or Wavelet transforms) for processing with 2D CNNs, studies^39^ have shown that 1D CNNs can extract high-quality features directly from raw signals. For instance, Kiranyaz et al.^39^ highlighted the effectiveness of 1D-CNNs, emphasizing their simplicity and reduced computational overhead compared to 2D counterparts. Moreover, this approach eliminates the need for domain-specific preprocessing or handcrafted features^30,40,41^, making it attractive for real-world deployment. With appropriate architectural tuning, a 1D-CNN can capture the relevant temporal patterns without transforming the data into other domains^32^. This design choice aligns with our broader goal of building a robust yet computationally lightweight system suitable for practical applications in road monitoring.

To further enhance the model’s ability to process multimodal sensor inputs, we employ a Multi-head CNN architecture, where each input source-such as IMU data from different vehicle mounting points-is processed independently through dedicated convolutional branches. Each input (e.g., signals from different IMU axes or sensor locations) has a dedicated set of filters, enabling specialized feature extraction tailored to each data stream. Although this design increases model complexity, it significantly enhances the network’s capacity to process heterogeneous sensor inputs effectively.

#### Recurrent neural networks

RNNs are designed for sequential data processing by retaining information from previous inputs, making them effective for time-series analysis^41^. However, they struggle with learning long-term dependencies due to the vanishing gradient problem. LSTM units address this limitation through gating mechanisms that regulate information flow, allowing the network to retain important data over longer sequences^32,41,42^. Their ability to capture both short- and long-term dependencies makes them well-suited for modeling complex time-dependent data.

In this study, LSTM layers are integrated into CNN and multi-head CNN architectures to enhance temporal pattern recognition in road surface classification tasks. The experimental design prioritizes lightweight, deployable solutions-an approach supported by the demonstrated efficiency and accuracy of LSTM-based architectures in similar vibration-based classification scenarios^32,41,42^. While attention mechanisms and transformer-based models have shown superior performance in capturing global sequence features, they generally require significantly larger datasets and greater computational resources^31,43^. These requirements make them less suitable for near real-time applications with limited training data, such as the one addressed in this study.

#### Base architectures

In the “1D-DL Architecture” section, from Fig. 5, an overview of the proposed base architectures for data processing is presented. Two main variants are explored: one relying only on convolutional layers and the other integrating convolutional layers with LSTM units to capture temporal dependencies, potentially improving classification performance. Both variants were tested on each of the constructed datasets, and all architectures output predictions regarding road surface conditions, classifying them into one of five categories: even road, uneven road, patch, manhole-pothole, and speed bump.

**Fig. 6:**
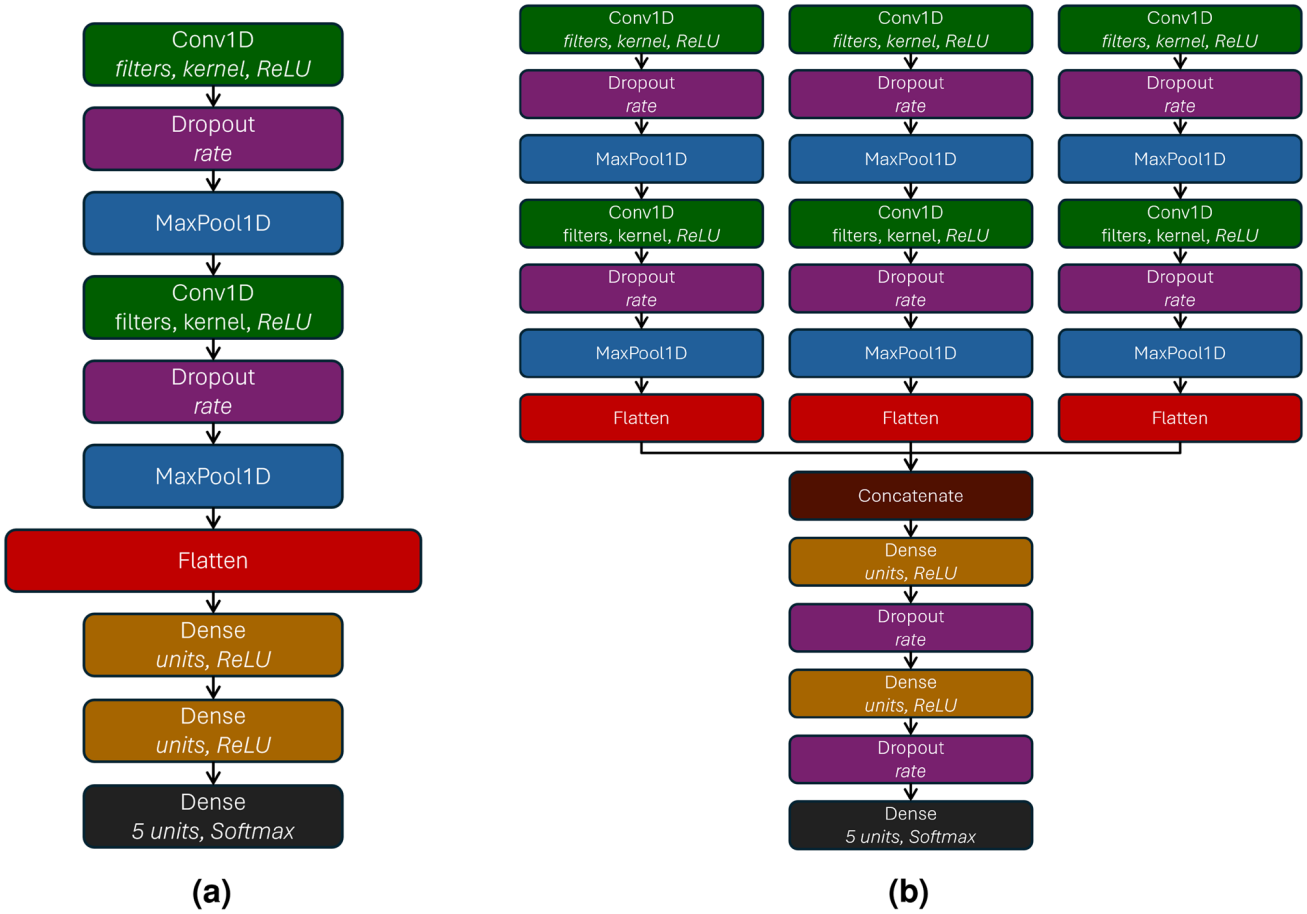
CNN architectures. **(a)** Single-head architecture. **(b)** Multi-head architecture.

The first architecture is based on convolutional layers. The architecture shown in Fig. 6afocuses on classifying road surfaces using datasets where IMU data are combined. The architecture uses the ReLU activation function to introduce non-linearity and enhance the network’s learning capacity. To combat overfitting, a dropout layer is applied after the convolutional layers. Following the initial convolutional operation, a MaxPool1D layer is used to downsample the feature maps, reducing dimensionality while retaining the most relevant features. A second Conv1D layer refines the feature extraction with additional filters, followed by another dropout layer. The output is then flattened and passed through two fully connected layers, which help the final decision-making process. The output layer employs a softmax activation function to produce multi-class predictions.

The second architecture, illustrated in Fig. 6b, utilizes a multi-head structure designed to process multiple data sources in parallel, making it well-suited for scenarios where inputs, such as data from different IMUs, show distinct characteristics. Unlike the previous architecture, where data from one or more IMUs are processed as a single sequence, this model uses separate convolutional heads to independently process the data from each IMU. Each head consists of a sequence of Conv1D layers, followed by dropout and MaxPooling layers, designed to extract features from the input source. Each head has two such convolutional sequences. After the feature extraction process, the flattened outputs from each head are concatenated into a feature vector. This concatenation preserves the distinct feature extraction performed by each head while combining the results for further processing. The merged feature vector is then passed through two dense layers with ReLU activation, each followed by dropout layers to mitigate overfitting. The final softmax-activated output layer generates the classification probabilities for the five road surface classes.

**Fig. 7:**
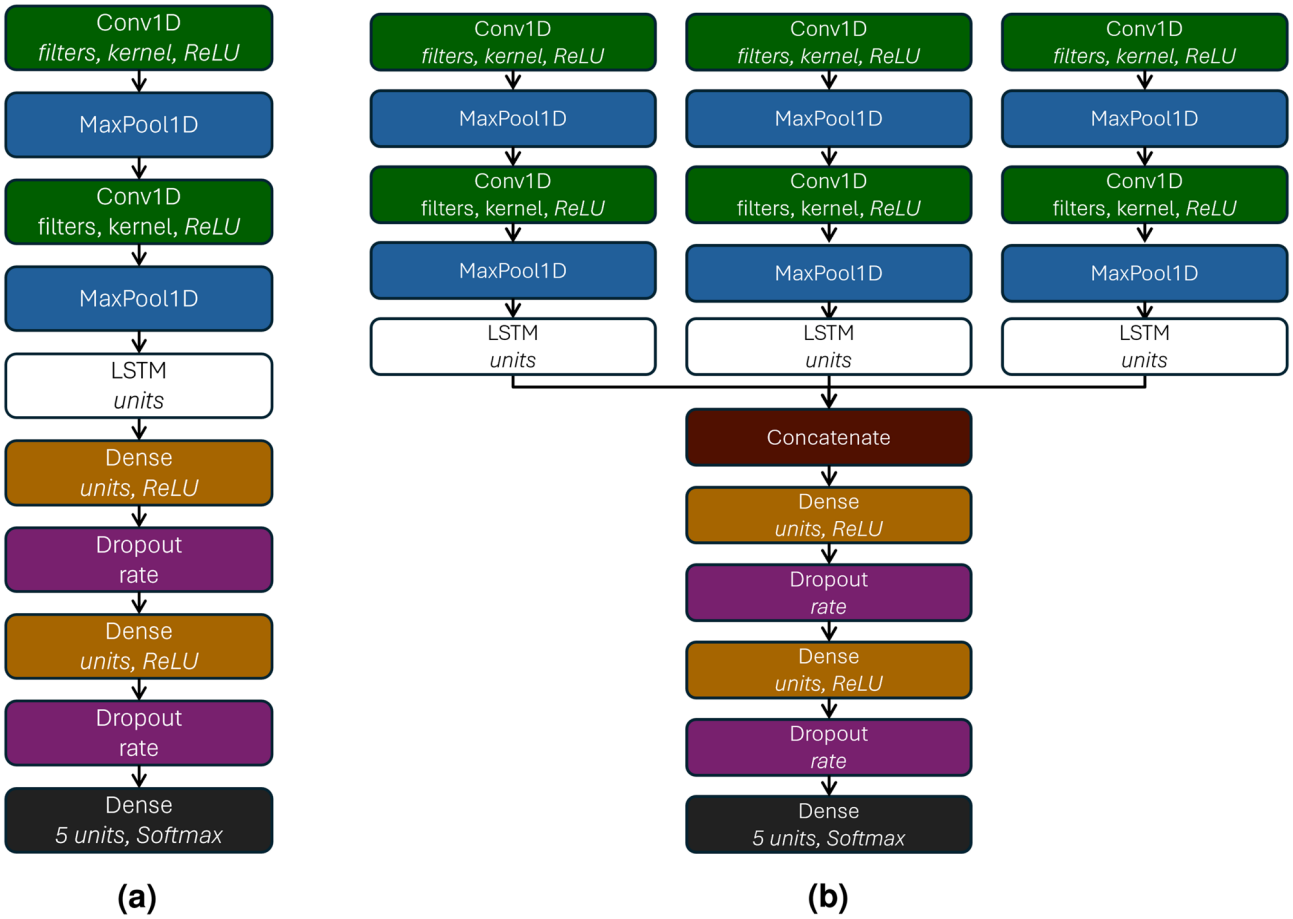
CNN+LSTM architectures. **(a)** Single-head architecture. **(b)** Multi-head architecture.

The architectures shown in Fig. 7 introduce LSTM units to capture temporal patterns within the data. While earlier models relied solely on convolutional layers for feature extraction, these hybrid architectures combine the strengths of both convolutional and recurrent layers.

In this design, convolutional layers serve as local feature extractors, transforming raw sensor inputs into higher-level representations, or feature maps^40^. Although some time-series models omit pooling to preserve fine-grained temporal details^40^, our experiments showed that MaxPooling improved performance. Pooling reduces the temporal dimension by retaining the most prominent activations, which helps suppress noise and emphasize features related to significant road events. This simplification also reduces the burden on the LSTM layers, allowing them to focus on modeling meaningful temporal patterns rather than minor fluctuations.

Following the convolution and pooling stages, the LSTM layers model the temporal evolution of the extracted features. By leveraging their memory capabilities, LSTMs capture dependencies across time and improve the network’s ability to interpret dynamic changes in the input^40^. Similar to Canizo et al.^41^, our model uses a windowed approach: each convolutional branch processes its respective time-series segment independently, producing a chronologically ordered sequence of feature maps. These sequences are then passed to the LSTM layers, preserving the temporal coherence of the original IMU signals throughout the network.

In the first architecture, illustrated in Fig. 7a, the structure resembles the previous single-head model, with two Conv1D layers for spatial feature extraction. However, instead of flattening the output and feeding it directly into dense layers, this architecture introduces an LSTM layer after feature extraction. Dropout is not applied between the convolutional layers, as the LSTM relies on maintaining consistent sequences to track temporal information. After the LSTM, the architecture concludes with two fully connected layers with ReLU activation, each followed by dropout to reduce overfitting.

The second architecture, shown in Fig. 7b, similar to the multi-head CNN architecture, processes data from multiple sensors independently, with each sensor stream handled by a separate head. Each head consists of two Conv1D layers followed by MaxPooling layers, just like in the multi-head CNN model. However, the key difference is that after pooling, each head includes an LSTM layer, enabling the model to capture how features evolve over time. After the LSTM layers, the output from each head is concatenated into a single feature vector. This combined representation is passed through two fully connected layers with ReLU activation, each followed by dropout to prevent overfitting. The final softmax-activated output layer provides the classification probabilities.

A key characteristic of the LSTM-based architectures is the absence of a flattening layer. Flattening the feature maps would convert the multidimensional data into a one-dimensional vector, resulting in the loss of the intrinsic structure and order of the temporal information. This alteration would restrict the LSTM’s ability to effectively model the relationships between data points across time steps, compromising its capacity to capture essential temporal dependencies.

The development of the proposed architectures was a gradual, iterative process rather than a one-time step. Initial models were tested on smaller subsets of the data to evaluate their early performance, allowing for incremental adjustments. Layers were added, removed, or rearranged based on these preliminary results to improve the architecture’s ability to capture relevant features while minimizing the risk of overfitting. During this process, key hyperparameters, such as the number of filters, kernel sizes, dropout rates, and learning rates, were systematically fine-tuned to enhance the model’s overall performance. Once the models showed promising results on smaller datasets, they were applied to the full dataset. This iterative refinement ensured that the final architectures were optimally suited for the task of road surface classification. Importantly, the validation dataset was used to adjust the models, rather than the test dataset, to prevent overfitting to the test data. This approach ensured that the final performance metrics truly reflected the models’ capabilities on unseen data, offering a realistic indication of their effectiveness in real-world scenarios.

## Experiment setup

After defining the base architectures and conducting preliminary tests for adjustments, the next step was to train the models following the methodology previously outlined. The architectures and model training were implemented in Python using Keras 2.10.0^44^. The training process was carried out on a system equipped with an Intel Core i7 12th generation processor, 32GB of RAM, and an NVIDIA RTX 3070 GPU.

### Classification performance metrics

Before diving into the training process, it is essential to clarify how the models were evaluated. The evaluation process is crucial for demonstrating the models’ ability to accurately identify and classify unseen data, highlighting their generalization capabilities. In the context of this study, it is important to note that the dataset is balanced, meaning that each class has an equal number of samples. This balance ensures that no class carries a specific weight, thereby preventing any distortion of the classification metrics due to unequal representation.

Given the balanced nature of the dataset, we employed the F1-score (equation 1) to assess the classification performance for each class individually. The F1-score, which is the harmonic mean of precision and recall, offers a balanced measure of a model’s ability to correctly classify positive instances while minimizing both false positives (precision) and false negatives (recall). This makes it particularly well-suited for evaluating models in tasks where both false alarms and missed detections are costly. By using the F1-score, we can highlight specific strengths and weaknesses in classification performance for each class, offering a subtle understanding of the model’s behavior.

To evaluate the overall performance across all classes, we used the macro-average F1-score (equation 2). Since our dataset is balanced, macro averaging assigns equal weight to each class, ensuring that poor performance in one class is not overshadowed by stronger performance in others. This is especially important in tasks where all classes are equally significant, as it ensures a more comprehensive assessment of model effectiveness.

1 $$\begin{aligned} \text {F1}_i = 2 \cdot \frac{\text {Precision}_i \cdot \text {Recall}_i}{\text {Precision}_i + \text {Recall}_i} \end{aligned}$$

2 $$\begin{aligned} \text {F1}_{\text {macro}} = \frac{1}{N} \sum _{i=1}^{N} \text {F1}_i \end{aligned}$$

### Model training

The training followed a grid search methodology to identify the optimal hyperparameters that deliver the best classification performance. This approach systematically evaluates multiple combinations of hyperparameters to determine the best-performing model based on the predefined classification metrics. Preliminary tests provided an approximation of the hyperparameters to be fine-tuned, as listed in Table 2.

**Table 2:** Hyperparameters used for testing the model configurations for both the CNN and CNN+LSTM architectures.

Hyperparameter	CNN Architecture	CNN+LSTM Architecture
# Filters - 1st Layer	16, 32, 64, 128	32, 64, 128
# Filters - 2nd Layer	16, 32, 64, 128	32, 64, 128
Kernel Size - 1st Layer	2, 3, 5	2, 3, 5
Kernel Size - 2nd Layer	2, 3, 5	2, 3, 5
Dropout rate	0.2, 0.5	0.2, 0.5
# LSTM Units	-	64, 128, 256, 512
# Neurons - 1st Layer	64, 128, 256	64, 128, 256
# Neurons - 2nd Layer	64, 128, 256	64, 128, 256
Epochs	30	30, 40

Training was conducted using the previously generated datasets, where 700 samples from each class were used for training and 200 samples from each class were used for validation. All models were trained using the Adam optimizer with a fixed learning rate of 0.001 and categorical cross-entropy as the loss function. A batch size of 32 was used across all training runs. The input shape varied depending on the dataset configuration, with a fixed window length of 216. The number of features changed based on the number of IMUs used and whether acceleration and/or angular velocity data were included.

The number of filters in the convolutional layers determines the model’s capacity to learn diverse features from the input data. Proper tuning of this hyperparameter is essential for maximizing the quality of feature extraction. Additionally, the size of the convolutional kernels influences the model’s ability to capture spatial patterns within the time-series data. To prevent overfitting, various dropout rates were tested to balance model complexity and generalization ability. This regularization technique helps ensure the model does not overfit the training data while maintaining strong classification performance on unseen data.

The number of neurons in the fully connected layers also impacts the model’s ability to learn high-level representations from the features extracted by the convolutional layers and LSTM units. Different neuron configurations were explored to optimize the model’s performance. The number of epochs was tuned to ensure sufficient training without overfitting, particularly for the CNN+LSTM architecture, where some configurations required more than 30 epochs to achieve satisfactory performance. For CNN+LSTM models, the number of LSTM units was adjusted to optimize the capture of temporal dependencies in the time-series data, adding complexity compared to purely convolutional architectures.

During the grid search, models were trained on the training subset of the dataset, and their performance was evaluated on the validation dataset using predefined classification metrics. After completing the grid search, the configuration that achieved the highest macro F1-score on the validation set was selected as the best model. This systematic approach ensured a thorough exploration of the hyperparameter space and helped identify the optimal combination for maximizing generalization to unseen data, which was tested in the next step of the experimental procedure.

## Results and analysis

### Models analysis

The CNN and CNN+LSTM architectures were evaluated using the optimal hyperparameters determined during the experimentation phase. The models’ classification metrics were obtained using the test data, comprising 10% of the entire dataset. Each model was evaluated using 100 samples from each class. This test data had never been used or seen by the models during training, providing a realistic approximation of the models’ performance in real-world scenarios.

Table 3 and Table 4 present the test results for the CNN and CNN+LSTM architectures. Each row corresponds to a specific architecture based on the dataset configuration-whether the input data was merged or split into multiple heads-and the features used, either combining acceleration and angular velocity in a single dataset or using them separately. The columns display the F1-scores achieved for each class, with the final column showing the macro F1-score for the entire model. The table highlights the best F1-Scores for each class (underlined), and the overall best score across all models is indicated in bold.

In the CNN models, summarized in Table 3, the highest overall performance was achieved by the single-head architecture using acceleration data from all three IMUs, with a macro F1-score of 0.9278. This model exhibited outstanding accuracy, particularly in classifying the even road and speed bump classes, outperforming all other configurations. However, like the other CNN-based models, it encountered challenges when classifying the uneven road and manhole-pothole classes, with average F1-scores of 0.8495 and 0.8629, respectively. The best-performing classes-Even Road, Patch, and Speed Bump-achieved F1-scores of 0.9135, 0.9458, and 0.9687, respectively.

A closer examination of the dataset variations reveals that models trained exclusively on sprung mass data (from the middle IMU) performed the worst, with an average F1-score of 0.8882. This underperformance can be attributed to the lack of information from the unsprung mass, which is crucial for capturing road surface irregularities more effectively. In contrast, models trained on unsprung mass data (Left and Right IMUs) showed better results, with average F1-scores of 0.912 and 0.9025 for the single-head and multi-head architectures, respectively. This suggests that unsprung mass data, from the wheels in contact with the road, plays a more significant role in accurately classifying the road surface conditions described in this study.

When both unsprung and sprung data were combined, the classification performance significantly improved. The single-head architecture using data from all three IMUs achieved an average F1-score of 0.9217 across its three variants, compared to 0.916 for the multi-head models. Although the difference is relatively small, this result indicates that merging data from multiple IMUs into a single input may be more effective than separating the data into distinct processing heads. This finding underscores the ability of convolutional layers to extract spatial features more robustly from combined datasets, particularly when dealing with acceleration data. While the performance gap between the architectures is minimal, it suggests that simpler architectures can sometimes outperform more complex ones, especially when the data is well-suited to unified feature extraction.

Notably, the best-performing model was not the one that used both acceleration and angular velocity but rather the one that relied solely on acceleration data. However, the multi-head approach that used both acceleration and angular velocity from the three IMUs still performed strongly, with an F1-score of 0.9234-only 0.0044 points behind the best model. This slight difference further highlights the capability of CNNs to process different sensor inputs effectively, while also suggesting that in certain cases, acceleration data alone may provide sufficient information for optimal classification performance.

**Table 3:** F1-scores for each class and macro F1-score obtained by the CNN models, using different dataset configurations.

**CNN Model** **IMU Data**	**F1-Score**					**Macro** **F1-Score**
	**Even Road**	**Uneven Road**	**Manhole-Pothole**	**Patch**	**Speed bump**	
Acc & Gyr Left-Middle-Right	0.9458	0.9	0.841	0.9469	0.9538	0.9175
Acc Left-Middle-Right	0.9505	0.8768	0.8854	0.9366	0.9899	**0.9278**
Gyr Left-Middle-Right	0.9412	0.8601	0.8571	0.9648	0.9751	0.9197
Acc & Gyr Left-Right	0.9163	0.8384	0.89	0.9703	0.9848	0.9199
Acc Left-Right	0.9126	0.84	0.8601	0.9366	0.9388	0.8976
Gyr Left-Right	0.9208	0.87	0.8725	0.949	0.9798	0.9184
Acc & Gyr Middle	0.8837	0.8163	0.8723	0.9703	0.9749	0.9035
Acc Middle	0.8972	0.8	0.8421	0.9333	0.9796	0.8904
Gyr Middle	0.8558	0.781	0.828	0.9347	0.9543	0.8707
Acc & Gyr Left & Right	0.9048	0.8543	0.8513	0.9353	0.9538	0.8999
Acc Left & Right	0.8848	0.8542	0.8763	0.9505	0.9744	0.9080
Gyr Left & Right	0.9082	0.8469	0.8469	0.932	0.9641	0.8996
Acc & Gyr Left & Middle & Right	0.9282	0.8731	0.875	0.9612	0.9796	0.9234
Acc Left & Middle & Right	0.9194	0.8558	0.8808	0.9406	0.9637	0.9121
Gyr Left & Middle & Right	0.9333	0.8756	0.8654	0.9246	0.9645	0.9127
**Average**	**0.9135**	**0.8495**	**0.8629**	**0.9457**	**0.9687**	**0.908**

Regarding the CNN+LSTM models, summarized in Table 4, the highest-performing model was trained on data from all three IMUs, incorporating both acceleration and angular velocity, achieving an overall macro F1-score of 0.9338. On the other hand, the models trained solely on sprung mass data (from the middle IMU) exhibited the poorest performance, with an average F1-score of 0.899. This suggests that sprung mass data alone is insufficient for accurately capturing the complexities of road surface classification.

In terms of class-wise performance, the even road, patch, and speed bump classes consistently yielded the highest classification accuracy, with average F1-scores exceeding 0.92 across all models. Contrarily, the uneven road and manhole-pothole classes presented the greatest challenges, with average F1-scores of 0.8561 and 0.8685, respectively, which negatively impacted the overall classification performance of the models.

When comparing models trained on unsprung mass data (from the left and right wheels), the single-head architecture slightly outperformed the multi-head architecture, with an average F1-score of 0.9278. However, the performance difference between the two architectures was minimal, indicating that both configurations were comparably effective in classifying road surface elements, despite the additional complexity of the multi-head design.

For models that used data from all three IMUs, the best-performing model was also the top model overall, with an F1-score of 0.9338. On average, the single-head models achieved an F1-score of 0.928, compared to 0.9162 for the multi-head models. Notably, the best multi-head model underperformed slightly compared to the top single-head model based on unsprung mass data alone. In both cases, the uneven road and manhole-pothole classes remained problematic, with F1-scores falling below 0.9, highlighting the difficulty in classifying these particular surface types.

**Table 4:** F1-scores for each class and macro F1-score obtained by the CNN+LSTM models, using different dataset configurations.

**CNN+LSTM Model** **IMU Data**	**F1-Score**					**Macro** **F1-Score**
	**Even Road**	**Uneven Road**	**Manhole-Pothole**	**Patch**	**Speed bump**	
Acc & Gyr Left-Middle-Right	0.9495	0.8922	0.8866	0.951	0.99	**0.9338**
Acc Left-Middle-Right	0.9394	0.8756	0.9036	0.9608	0.97	0.9299
Gyr Left-Middle-Right	0.9326	0.8612	0.8615	0.9608	0.9849	0.9202
Acc & Gyr Left-Right	0.934	0.8945	0.898	0.9275	0.9851	0.9278
Acc Left-Right	0.9023	0.8526	0.8922	0.934	0.9485	0.9059
Gyr Left-Right	0.9146	0.8585	0.8687	0.9447	0.9849	0.9143
Acc & Gyr Middle	0.9032	0.7933	0.8187	0.9252	0.9848	0.885
Acc Middle	0.9036	0.8213	0.8235	0.9137	0.9744	0.8873
Gyr Middle	0.9126	0.8223	0.829	0.9463	0.9849	0.899
Acc & Gyr Left & Right	0.9091	0.87	0.8821	0.9645	0.9849	0.9221
Acc Left & Right	0.9038	0.8454	0.8878	0.9208	0.97	0.9056
Gyr Left & Right	0.9307	0.8762	0.8723	0.934	0.9754	0.9177
Acc & Gyr Left & Middle & Right	0.9347	0.8763	0.8756	0.9565	0.995	0.9276
Acc Left & Middle & Right	0.92	0.8416	0.8731	0.94	0.9751	0.91
Gyr Left & Middle & Right	0.932	0.8601	0.8542	0.9282	0.98	0.9109
**Average**	**0.9215**	**0.8561**	**0.8685**	**0.9405**	**0.9792**	**0.9131**

**Fig. 8:**
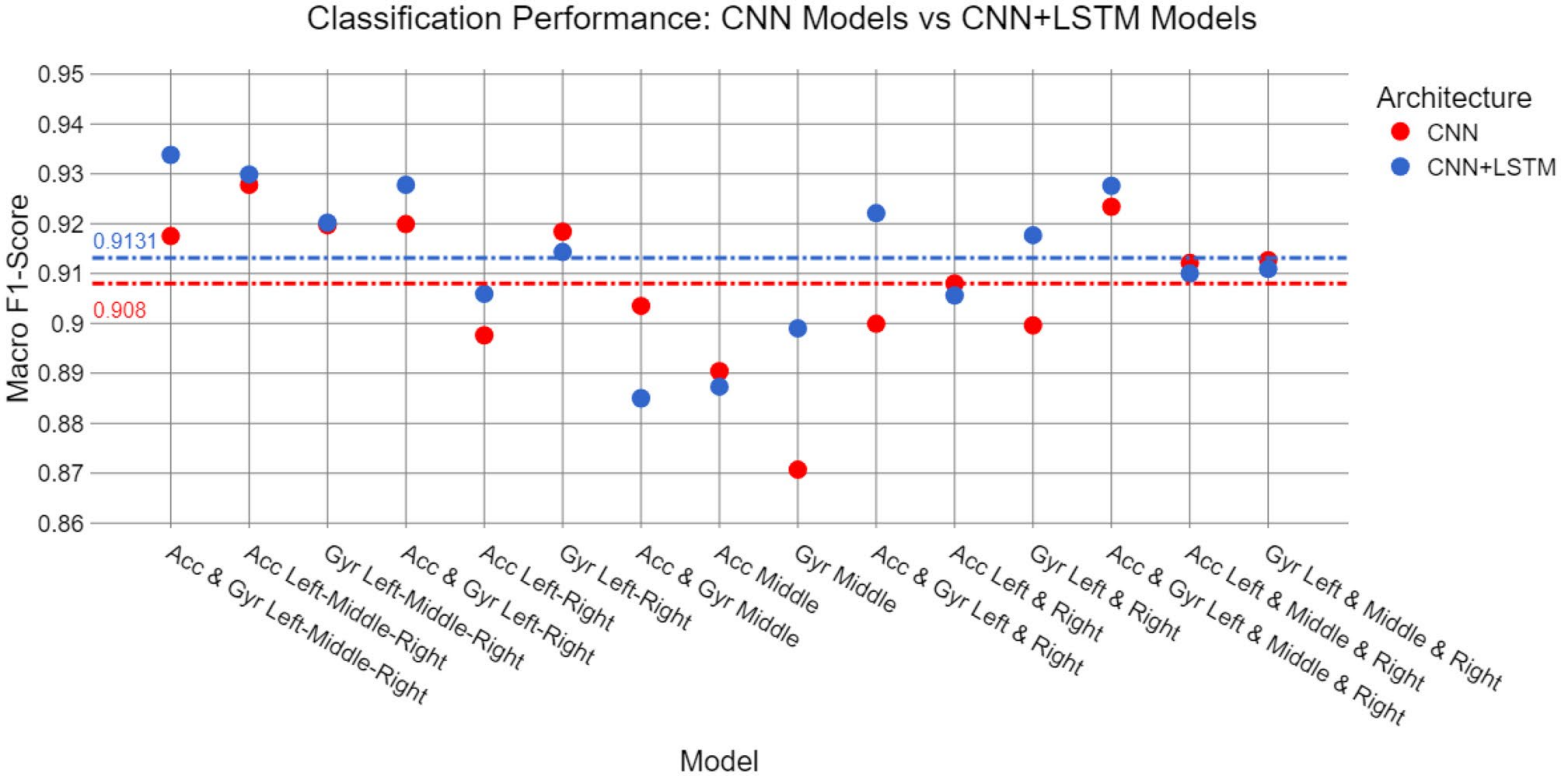
Comparison of the macro F1-score obtained by the CNN and CNN+LSTM model variants.

As shown in Fig. 8, the architectures combining CNN and LSTM layers (highlighted in blue) generally outperformed those based only on CNNs (highlighted in red). On average, the CNN+LSTM models achieved a macro F1-score of 0.9131, compared to 0.908 for the CNN-only models. This improvement suggests that the LSTM layers provide a significant advantage by capturing temporal dependencies in the data.

However, there are some important observations. Although the CNN+LSTM models performed better in most cases, certain CNN-only models outperformed them, such as the model trained on middle IMU data that used acceleration and gyroscope data, and only acceleration data. Moreover, the overall performance difference between the two architectures was relatively small in most cases, except for two notable instances. The CNN model based on middle IMU gyroscope data showed a significant performance drop compared to its CNN+LSTM counterpart. Similarly, the multi-head CNN model using unsprung mass data (Left and Right IMUs) underperformed when using both acceleration and angular velocity data, as well as when using angular velocity alone.

At the center of Fig. 8, it is clear that models trained exclusively on sprung mass data (Middle IMU) delivered the worst performance. While further training and testing are required for a definitive conclusion, the results from this study suggest that sprung mass data alone is insufficient for effective classification. Across both architectures and various datasets, these models failed to match the performance of models trained on unsprung mass or combined datasets.

For models trained on unsprung mass data (Left and Right IMUs), the best results were obtained by combining acceleration and angular velocity data, followed by using gyroscope data. The results indicate that acceleration data alone from the unsprung mass presents more difficulty for road surface classification, regardless of whether a single-head or multi-head architecture is used. In both cases, the combination of acceleration and angular velocity data consistently led to better performance.

When comparing models that utilized data from all three IMUs, those that processed the data as a single dataset outperformed models that separated the data into multiple heads, regardless of whether CNN or CNN+LSTM architectures were used. This finding suggests that the multi-head approach generally underperformed compared to single-head architectures, reinforcing the idea that simpler models can sometimes offer superior classification capabilities, particularly when leveraging combined data inputs.

**Fig. 9:**
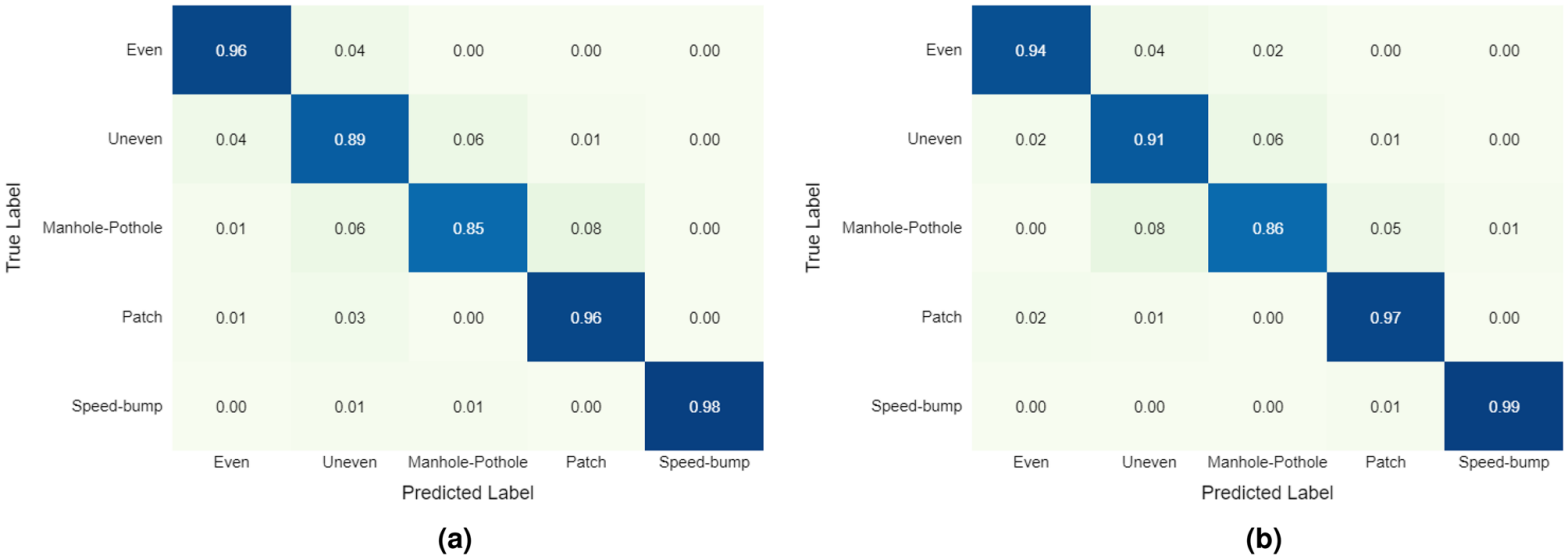
Confusion matrices of the best-performing models of each architecture type. **(a)** CNN model based on Left-Middle-Right acceleration data. F1-score: 0.9278. **(b)** CNN+LSTM model based on Left-Middle-Right acceleration and angular velocity data. F1-score: 0.9338.

Table 5 and Fig. 9 present the set of hyperparameters and the confusion matrices used for the best-performing models from each architecture, tested on 100 samples per class. Figure 9ashows the confusion matrix for the CNN-only model, which achieved a macro F1-score of 0.9278, while Fig. 9bdisplays the results for the CNN+LSTM model, which reached an F1-score of 0.9338. Although both models performed similarly overall, there are some notable differences.

Both models faced challenges in classifying the uneven road and manhole-pothole classes. However, the CNN+LSTM model demonstrated slightly better performance in these categories. In contrast, the CNN-only model surpassed the even road class in classification, outperforming the CNN+LSTM model. The confusion matrices also reveal that the manhole-pothole class was often misclassified and frequently confused with the uneven road and patch classes. Additionally, the uneven road class was sometimes incorrectly identified as either even road or manhole-pothole. These misclassifications suggest a potential area for improvement by refining the model’s ability to differentiate between these similar classes. Addressing these issues could involve providing more diverse and representative examples during training to reduce cross-class confusion.

On the other hand, the speed bump class consistently achieved exceptional performance, with minimal misclassification across both models. This demonstrates the model’s robustness in identifying speed bumps, likely due to the distinct and easily recognizable features associated with this class.

**Table 5:** Hyperparameters of the best-performing models of each architecture.

**Hyperparameter**	**CNN Model** **Left-Middle-Right** **Acc**	**CNN+LSTM** **Left-Middle-Right** **Acc & Gyr**
# Filters - 1st Layer	64	64
# Filters - 2nd Layer	128	128
Kernel Size - 1st Layer	2	3
Kernel Size - 2nd Layer	2	3
Dropout rate	0.5	0.2
# LSTM Units	-	256
# Neurons - 1st Layer	128	64
# Neurons - 2nd Layer	64	128
Epochs	30	30
Macro F1-Score	**0.9278**	**0.9338**

### Inference speed analysis

The inference time of each model was measured (in milliseconds) using its corresponding sensor configuration. All experiments were conducted on a computer equipped with an Intel(R) Core(TM) i9-14900HX CPU, 96 GB RAM, and an NVIDIA GeForce RTX 4070 GPU with 8 GB GDDR6 VRAM. All models were implemented using Keras 2.10.0, and each configuration was tested on 500 samples from the test dataset. Inference time was computed as the average time per prediction.

As shown in Table 6, CNN-based models exhibited low inference times across all configurations. The fastest inference was achieved using gyroscope data from the left, middle, and right IMUs, implemented in a single-head architecture, with a mean of 28.63 ms and a standard deviation of 1.88 ms. The slowest configuration involved accelerometer and gyroscope data from all IMUs, arranged in a three-head architecture, with a mean inference time of 33.31 ms.

**Table 6:** Inference speed (in milliseconds) for each sensor configuration using the CNN models.

**IMU Data**	**Mean (ms)**	**STD (ms)**
Gyr Left-Middle-Right	28.63	1.88
Gyr Middle	28.79	1.37
Gyr Left-Right	28.84	1.42
Acc Middle	28.92	1.42
Acc & Gyr Middle	28.93	1.69
Acc & Gyr Left-Right	29.12	1.50
Acc Left-Middle-Right	29.16	1.54
Acc & Gyr Left-Middle-Right	29.31	2.36
Acc Left-Right	29.42	2.21
Acc & Gyr Left & Right	30.65	1.52
Gyr Left & Right	31.75	2.23
Acc Left & Right	31.81	1.71
Gyr Left & Middle & Right	32.59	1.64
Acc Left & Middle & Right	32.97	1.22
Acc & Gyr Left & Middle & Right	33.31	1.89

Adding LSTM layers increases the temporal modeling capacity of the architecture and its classification performance, but it also introduces additional latency. As shown in Table 7, the lowest inference time was observed for configurations using gyroscope or accelerometer data from the middle IMU, both achieving a mean of 31.38 ms. Contrarily, the highest latency occurred when using gyroscope data from all positions, deployed over a three-head architecture, with a mean inference time of 48.39 ms and a standard deviation of 6.59 ms.

While CNN+LSTM configurations generally required more time, several still completed inference in under 34 ms, indicating potential for real-time deployment, particularly in systems where temporal context is crucial.

**Table 7:** Inference speed (in milliseconds) for each sensor configuration using the CNN+LSTM models.

**IMU Data**	**Mean (ms)**	**STD (ms)**
Gyr Middle	31.38	1.44
Acc Middle	31.38	1.18
Acc & Gyr Middle	32.00	1.12
Acc & Gyr Left-Right	32.05	2.11
Acc Left-Right	33.04	1.51
Gyr Left-Middle-Right	33.10	1.28
Acc Left-Middle-Right	33.33	1.35
Acc & Gyr Left-Middle-Right	33.80	1.77
Gyr Left-Right	36.69	1.78
Acc Left & Right	37.23	1.39
Acc & Gyr Left & Right	37.63	3.15
Gyr Left & Right	39.37	2.00
Acc Left & Middle & Right	45.03	4.78
Acc & Gyr Left & Middle & Right	45.39	5.48
Gyr Left & Middle & Right	48.39	6.59

These findings highlight the trade-off between architectural complexity and real-time feasibility. Lightweight CNN architectures offer faster inference, while CNN+LSTM configurations can model temporal dependencies more effectively at the cost of increased latency. Selecting the optimal configuration depends on the hardware, timing restrictions, and desired predictive performance of the RSM system.

### Comparison analysis

It is important to compare this study’s results with other research focused on classifying road surface conditions using similar methodologies. However, several factors must be considered when comparing studies. First, different studies use varying metrics to report their models’ performance, and these metrics are often heavily influenced by the robustness and balance of the datasets used. This makes it challenging to directly compare results, as metrics reported from unbalanced datasets might obscure poor performance in certain classes. For this reason, we report the macro F1-score, a widely accepted metric that considers performance across all classes, even though our dataset is balanced.

Additionally, the type and number of classes differ across studies, further complicating direct performance comparisons. Despite this, we provide both a detailed class-by-class comparison with a similar study (Table 8) and a summary comparison with other related works (Table 9). The latter highlights relative performance and methodological differences to contextualize the contributions of our work.

**Table 8:** Comparison of our best model, against model proposed by Raslan et al.^32^.

**Classes**	**Our model**	**Raslan et al.**
Even Road	0.9495	0.9569
Uneven Road	0.8922	0.9053
Manhole-Pothole	0.8866	0.9476
Patch	0.9510	-
Speed bump	0.99	0.925
Macro F1-score	**0.9338**	**0.9337**

**Table 9:** Comparison with related studies in terms of task scope, classification performance, and methodology. Due to differences in class definitions and evaluation metrics, the results are not directly comparable but serve to contextualize the contributions of this study.

**Study**	**Classes**	**Metric Used**	**Result**	**Notes**
**This study**	Even, Uneven, Manhole-Pothole, Patch, Speed bump	Macro F1-score	0.9338	Deep learning, raw IMU signals
Raslan et al.^32^	Normal, Potholes, Speed bump, Bad	Macro F1-score	0.9337	Feature engineering on IMU data
Hijji et al.^30^	Normal road, Pothole	F1-score	0.899	Multi-head CNN, fusion of image and IMU
Celaya-Padilla et al.^45^	Speed bump detection	Accuracy	0.9714	Genetic algorithm with statistical features
Shtayat et al.^46^	Alligator cracks, Edge cracks, Longitudinal cracks, Patches and alligator cracks, Patches	F1-score (Patch)	0.96	Traditional ML (SVM) on curated dataset

The first comparison is with the study by Raslan et al.^32^, which utilized a single IMU to capture the vehicle’s response to road surface conditions, applying CNNs combined with RNN layers for classification. As shown in Table 8, the results are remarkably similar to ours, with both studies achieving comparable macro F1-scores. A key distinction lies in the data preparation phase: while Raslan et al. employed feature engineering to preprocess the raw IMU data, our approach directly used the raw IMU readings. Additionally, our classification includes the Patch class, which was not considered in their study. While our model slightly underperformed in the Manhole-Pothole category, it showed improved performance for the Speed bump class.

Further comparisons are summarized in Table 9. Hijji et al.^30^ integrated vibration and image data using a multi-head CNN and achieved an F1-score of 0.899 for binary classification of normal roads versus potholes. While their overall performance was slightly lower than ours, their integration of image data with IMU readings highlights the potential for multimodal data fusion to enrich classification performance, providing a valuable perspective for future work on improving road surface monitoring. Celaya-Padilla et al.^45^ focused on detecting speed bumps using statistical features and genetic algorithms, achieving an accuracy of 97.14%, which compares to our 0.99 F1-score for the same class. Shtayat et al.^46^ used Support Vector Machines to classify road surface distresses, obtaining an F1-score of 0.96 for the Patch class, which is comparable to our 0.951. This indicates that conventional machine learning approaches, when paired with well-curated datasets, can still deliver competitive results, even alongside more sophisticated deep learning techniques. Although the metrics and class labels vary across these studies, the comparison highlights that our approach remains competitive, even with a more complex multi-class classification task and without relying on handcrafted features.

Furthermore, to evaluate the effectiveness of our proposed architecture, we conducted a comparative experiment using the architecture proposed by Raslan et al.^32^. Two variants were tested: (a) to assess whether, when trained on similar data to that used in the original study (i.e., only the Middle IMU), the architecture could achieve results comparable to ours, and (b) to determine whether increasing the quantity of input data-by including signals from all three IMUs (Left, Middle, Right)-could enhance the performance of the model.

The first variant was trained using only the 6 features from the Middle IMU, while the second variant was trained using the 18 features from the LMR dataset. In both cases, the datasets were transformed into the frequency and time–frequency domains, in line with the input processing described in Raslan’s study. The models were trained using the same hyperparameters, but early stopping (patience = 5) was included to avoid overfitting.

**Fig. 10:**
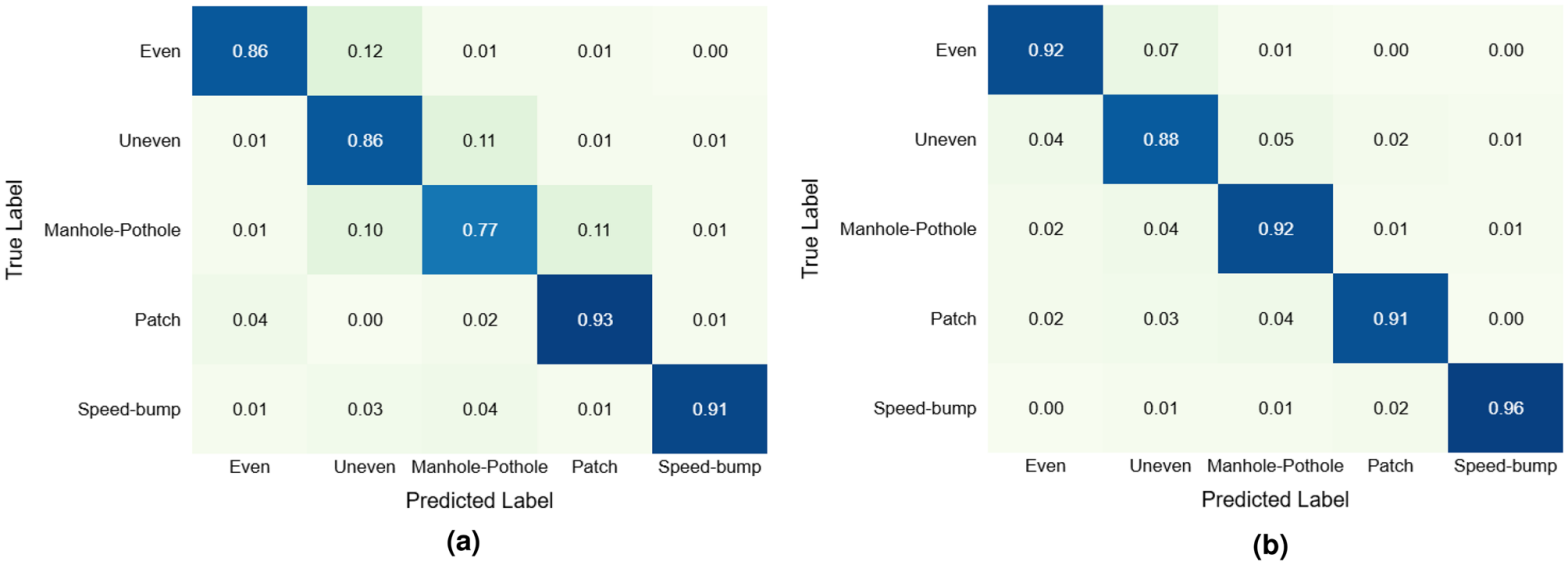
Confusion matrices of the models trained with the architecture proposed by Raslan et al.^32^. **(a)** The model was trained with the 6 features from the Middle IMU, similar to the dataset proposed by Raslan et al. F1-score: 0.8666. **(b)** The model was trained with the 18 features from the LMR dataset. F1-score: 0.9183.

The confusion matrices in Fig.10 illustrate the performance of both models: the left matrix corresponds to the Middle IMU model, and the right matrix to the LMR dataset model. The LMR model achieved a macro F1-score of 0.9183, while the Middle-only model reached 0.8666. These results confirm that, although adding more input data does improve Raslan’s model performance, it still does not reach the accuracy of our best-performing architecture. This comparison highlights the robustness of our approach (Fig. 9b), which introduces architectural improvements that lead to better generalization and classification performance.

## Conclusion

This study presented a comprehensive comparative analysis of deep learning architectures for road surface classification using on-board inertial measurements. Five road surface types-even road, uneven road, manhole-pothole, patch, and speed bump-were distinguished using thirty deep learning models, built from different combinations of convolutional and LSTM layers, and trained on various configurations of acceleration and angular velocity data collected from both sprung and unsprung masses of a vehicle.

The results revealed the limitations of purely convolutional architectures in modeling temporal patterns in time-series data. In contrast, CNN+LSTM architectures demonstrated a clear advantage in capturing temporal dependencies, leading to improved classification performance. A key insight was the superior performance of models trained on fused data from both sprung and unsprung masses, highlighting the importance of sensor fusion for accurate road surface classification. However, the influence of sensor placement, vehicle dynamics (e.g., mass and speed), and road conditions suggests the need for further validation across a broader dataset.

Importantly, the findings also showed that high performance can be achieved using raw IMU data without explicit feature engineering, indicating the potential for simplified preprocessing pipelines. Nevertheless, the inclusion of engineered features remains a promising avenue to explore for enhancing model accuracy.

Future work should investigate more sophisticated single- and multi-head architectures, examine the impact of individual sensor axes on classification outcomes, and evaluate the system under real-time deployment conditions. Incorporating image data from on-board cameras may further improve robustness. A hybrid approach that combines inertial and visual information, possibly augmented with semi-supervised or self-learning techniques, could provide a more adaptive and reliable solution for road surface monitoring in real-world conditions.

## Data Availability

The datasets generated and analyzed during the current study are available from the corresponding author upon reasonable request.
